# Supplementary material for: Uncovering miRNA-mRNA Regulatory Modules in Developing Xylem of Pinus massoniana via Small RNA and Degradome Sequencing
Source: Int J Mol Sci. 2021 Sep 21;22(18):10154. doi: 10.3390/ijms221810154 (PMC8472836; doi:10.3390/ijms221810154)
Supplement: Supplementary file 1 [file ijms-22-10154-s001.zip › ijms-1365557-supplementary/Additional file S2.zip!/Additional file 2/pma-miR946a-3p_Cluster-4562.1_426_TPlot.pdf]

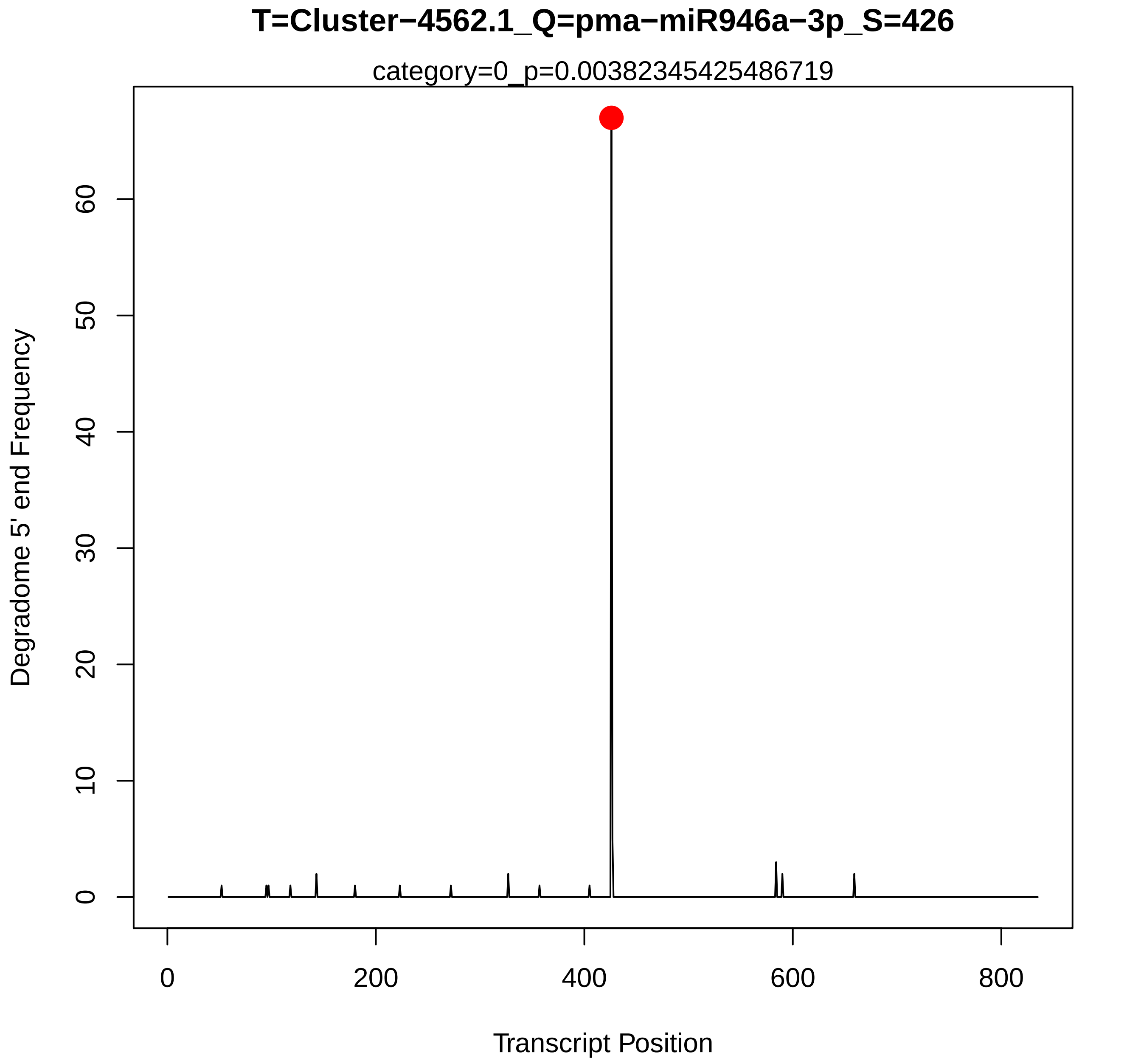
T=Cluster−4562.1_Q=pma−miR946a−3p_S=426
category=0_p=0.00382345425486719
Degradome 5' end Frequency
0
10
20
30
40
50
60
0
200
400
600
800
Transcript Position
